# Quantification of HLA-DM-Dependent Major Histocompatibility Complex of Class II Immunopeptidomes by the Peptide Landscape Antigenic Epitope Alignment Utility

**DOI:** 10.3389/fimmu.2018.00872

**Published:** 2018-05-03

**Authors:** Miguel Álvaro-Benito, Eliot Morrison, Esam T. Abualrous, Benno Kuropka, Christian Freund

**Affiliations:** ^1^Protein Biochemistry, Institute for Biochemistry, Freie Universität Berlin, Berlin, Germany; ^2^Computational Molecular Biology Group, Institute for Mathematics, Freie Universität Berlin, Berlin, Germany

**Keywords:** major histocompatibility complex of class II immunopeptidome, HLA-DM expression, nested peptides, register shifts, label-free quantification

## Abstract

The major histocompatibility complex of class II (MHCII) immunopeptidome represents the repertoire of antigenic peptides with the potential to activate CD4^+^ T cells. An understanding of how the relative abundance of specific antigenic epitopes affects the outcome of T cell responses is an important aspect of adaptive immunity and offers a venue to more rationally tailor T cell activation in the context of disease. Recent advances in mass spectrometric instrumentation, computational power, labeling strategies, and software analysis have enabled an increasing number of stratified studies on HLA ligandomes, in the context of both basic and translational research. A key challenge in the case of MHCII immunopeptidomes, often determined for different samples at distinct conditions, is to derive quantitative information on consensus epitopes from antigenic peptides of variable lengths. Here, we present the design and benchmarking of a new algorithm [peptide landscape antigenic epitope alignment utility (PLAtEAU)] allowing the identification and label-free quantification (LFQ) of shared consensus epitopes arising from series of nested peptides. The algorithm simplifies the complexity of the dataset while allowing the identification of nested peptides within relatively short segments of protein sequences. Moreover, we apply this algorithm to the comparison of the ligandomes of cell lines with two different expression levels of the peptide-exchange catalyst HLA-DM. Direct comparison of LFQ intensities determined at the peptide level is inconclusive, as most of the peptides are not significantly enriched due to poor sampling. Applying the PLAtEAU algorithm for grouping of the peptides into consensus epitopes shows that more than half of the total number of epitopes is preferentially and significantly enriched for each condition. This simplification and deconvolution of the complex and ambiguous peptide-level dataset highlights the value of the PLAtEAU algorithm in facilitating robust and accessible quantitative analysis of immunopeptidomes across cellular contexts. *In silico* analysis of the peptides enriched for each HLA-DM expression conditions suggests a higher affinity of the pool of peptides isolated from the high DM expression samples. Interestingly, our analysis reveals that while for certain autoimmune-relevant epitopes their presentation increases upon DM expression others are clearly edited out from the peptidome.

## Introduction

Major histocompatibility complex of class II (MHCII) molecules are expressed in professional antigen-presenting cells (APCs) and present epitopes derived primarily from extracellular antigens to CD4^+^ T cells (1). T cells sense the presence of antigenic peptides in the context of the corresponding peptide–MHCII complex (pMHCII) *via* their T cell receptor (TcR) and the CD4 co-receptor. Engagement of pMHCII complexes by TcR–CD4 and the supporting interactions of co-stimulatory molecules trigger activation of T cells. Initial *in vitro* studies addressing the influence of the density of pMHCII complexes at the surface of the APC revealed that approximately 50–200 pMHCII complexes were sufficient to trigger T cell activation (2, 3). However, this number clearly depends on the APC cell type (3) and the specific TcR–pMHCII pair under consideration (4). Regardless of the minimum number of pMHCII complexes required at the cell surface to trigger stimulation of T cell clones, the pMHCII density influences the process of Th1/Th2 differentiation (5). More recently, the pMHCII density has also been correlated to CD4^+^ T cell differentiation into Tregs (6, 7).

One major challenge when assessing the composition and density of both pMHCI and pMHCII complexes at the cell surface is the lack of unbiased methods that allow for the direct and global quantification of peptide presentation, as recently reviewed by Purcell et al. (8). The density and presentation of specific pMHC complexes at the cell surface is most often analyzed by flow cytometry or indirectly as a response to titrations of specific antigens to restricted T cell hybridomas in cell culture. However, these methods require antigen-specific reagents and are reported to exhibit high variability and low reproducibility in measurements across different labs (8). Mass spectrometric analysis of the immunopeptidome associated with MHC molecules, on the other hand, has advanced significantly in the last decade, allowing higher-resolution measurements and the deconvolution of complex peptide samples with fewer requirements for sample preparation.

Quantitative proteomic approaches have been used successfully for the analysis of complete MHC immunopeptidomes and can be coupled to quantification methods based on Stable Isotope Labeling by Amino Acids in Cell Culture (SILAC) (9, 10) or Absolute QUAntification (AQUA), which uses spiked-in isoto-pically labeled peptides; these methods have been applied to both shotgun and targeted approaches based on Selected or Multiple Reaction Monitoring (SRM/MRM) (11, 12). The main inconvenience of using SILAC for analyzing the MHCII immunopeptidome is that it does not allow for labeling of primary cells or clinically relevant tissue samples due to the requirement for incorporation of labeled amino acids in cell culture. In addition, there is no specific cleavage during peptide processing by cathepsins, so labeled residues (e.g., Lys or Arg in the case of tryptic digest) are not guaranteed at a fixed number per peptide. While the repertoire for cleaved sites could be expanded by the use of additional proteases like elastase, this would require additional isotopically labeled amino acids and would still not mimic the unspecific cleavage of cathepsins. For AQUA-based approaches, standard peptides must be defined *a priori*, limiting the identification of novel epitopes. Recently, Bergseng et al. (13) made use of label-free quantification (LFQ) to determine the endogenous immunopeptidome associated with HLA-DQ molecules (13). Finally, quantification of peptides by chemical isobaric tags like the tandem mass tag system or isobaric tags for relative and absolute quantitation (iTRAQ) has also been used in case of MHC immunopeptidomes (14) although it results in considerable expenses. While this could be a fruitful avenue of development in the future, the current state-of-the-art of LFQ offers the best balance of robust and cost-effective quantification of immunopeptidome analysis in, e.g., clinical settings.

Peptides eluted from MHCII molecules are of variable length, usually between 11 and 25 amino acids, with only 9 of these residues defining the core binding motif. The N- and C- terminal extensions can also impact the affinity of peptides to MHCII molecules (albeit to a lower degree than the consensus epitope), which expands the optimal antigen size to a length of 13 amino acids (15). Variable length arises in many cases from peptides belonging to series of nested peptides, which result from trimming of the N- and C-termini by exoproteases (16). When these nested peptides are quantified individually by MS, information about the abundance of their shared consensus binding motif is then obscured. In this context, the development of LFQ-based approaches to group and quantify nested consensus epitopes would help to overcome such limitations (17). Here, we introduce a Python algorithm to define sets of nested peptides from MHCII-eluted peptides identified and quantified by MS. We apply this algorithm to determine the impact of different expression levels of the MHCII master peptide editor HLA-DM (18, 19) on the immunopeptidome presented by HLA-DR3. Nested peptides are used to retrieve the core antigenic sequences based on *per*-residue summed intensities. Subsequently, the identified epitopes are quantified based on relative intensity of the nested peptides. With this approach, we can show that the relative expression levels of HLA-DM affect the overall composition of the HLA-DR immunopeptidome in a qualitative and quantitative manner. Moreover, our approach could be easily adopted to study quantitative differences between immunopeptidomes in other cellular or organismic contexts by LFQ.

## Materials and Methods

### Cell Lines and Flow Cytometry

Constructs based on the lentiviral vector LeGO-iG2 (20) were used to pack lentiviral particles in HEK293T cells upon transfection with pMDLg, pRSV-Rev, and pMD2.G (vsv-g). HEK293T cells were grown in DMEM with 5% FCS in the presence of 5% CO_2_, and were transfected with PEI. Viruses were harvested after 48 h and used for spinoculation of target cell lines (see below) using 1,200 × *g* during 45 min at 30°C in the presence of 8 µg/ml Polybrene. After 72 h cells were expanded and sorted according to GFP expression levels.

T2 cell lines stably expressing HLA-DR3 were grown in IMDM with 10% FCS at 37°C in the presence of 5% CO_2_. This cell line was transduced with lentivirus bearing cDNA constructs for *DMB* and *DMA* genes spaced by a sequence encoding for a T2A peptide. Transduced T2-DR3 cells were sorted based on GFP expression levels and single cell clones were isolated and expanded.

Cells (5 × 10^5^) in mid-log growth phase were washed and probed (as indicated by the vendor) for HLA-DR (L243—BioLegend), class II invariant chain peptide (CLIP) (CerCLIP.1—Santa Cruz) or HLA-DM (MaP.DM1—BioLegend). A secondary anti-mouse PE-conjugated antibody (BioLegend) was used for detection. Intracellular staining was performed using the Cytofix-cytoperm kit (BD). Measurements were performed in a Canto II flow cytometer (BD) and analyzed using FCS express 6.0 (De Novo software).

### SDS-Stable Dimer Assay for Stable Cell Lines

Cells (10^7^) were harvested, washed twice in cold PBS, and snap-frozen in liquid N_2_. Pellets were resuspended in 500 µl of lysis buffer (buffer A: Tris–HCl 50 mM pH 8.0, NaCl 150 mM, plus 1% Triton X-100, 1:20 cOmplete protease inhibitor) for 30 min. Cell lysates were cleared by centrifugation at 15,000 × *g* for 30 min, and the supernatants were collected for subsequent tests. From each cell line, 20 µg of cell lysate was diluted in loading buffer (with 100 mM DTT) and left at RT (unboiled) or boiled for 5 min. Afterward, the samples were loaded and resolved on a 10% SDS-PAGE gel. Proteins were then transferred to a nitrocellulose membrane and stained for different antibodies as indicated. L243 was used at a 1 µg/ml dilution to detect SDS-stable dimers, 1B5 (Abcam) was used at a 0.5 mg/ml dilution to detect HLA-DRA under denaturing conditions, and the loading control was anti-β-actin-HRP (Abcam), used at a 1:50,000 dilution. Signals were detected using luminol and a chemiluminescence–fluorescence imager (ChemoCam HR 16-3200, Intas GmbH). Relative quantification of SDS-stable dimer formation was analyzed using the Chemostar software provided by the vendor. Both dimer and DRA signals were made relative to the loading control, and then the ratio between the two signals was calculated. Three independent cell cultures were analyzed twice each in independent western blots.

### Peptide–MHCII Complex Isolation and Sample Preparation for Mass Spectrometry

Biological replicates (2 × 10^8^ cells) were grown in independent cultures, harvested and snap-frozen in liquid nitrogen in three aliquots constituting the different technical replicates. Cell lysis was performed in an end-over-end rotator in the presence of lysis buffer (buffer A: Tris–HCl 50 mM pH 8.0, NaCl 150 mM, plus 1% CHAPS, 1:20 cOmplete protease inhibitor) for 1 h. Cell lysates were cleared by centrifugation at 15,000 × *g* for 30 min, and the supernatants were collected for subsequent pMHCII purification. Purification of pMHCII from the corresponding supernatants was performed using immunoaffinity chromatography with the L243 antibody coupled to FF-Sepharose. Beads were washed with 10 volumes of buffer A with 500 mM NaCl, 10 volumes of lysis buffer with no NaCl, 10 volumes of buffer A with 150 mM NaCl, and finally 10 volumes of H_2_O. MHCII and peptides were dissociated from each other and from the column by adding 5 volumes of TFA to 0.02%. The resulting peptide mixtures were fractionated using a 10 kDa cutoff micro-spin filter device and washed using C18 zip-tips.

### Mass Spectrometry

Peptides were reconstituted in 20 μL of 0.1% (v/v) TFA, 5% (v/v) acetonitrile, and 6.5 µL were analyzed by a reversed-phase capillary nano liquid chromatography system (Ultimate 3000, Thermo Scientific, USA) connected to an Orbitrap Velos mass spectrometer (Thermo Scientific). LC separations were performed on a capillary column (Acclaim PepMap100 C18, 2 μm, 100 Å, 75 μm i.d. × 25 cm, Thermo Scientific) at an eluent flow rate of 300 nL/min. Mobile phase A contained 0.1% formic acid in water, and mobile phase B contained 0.1% formic acid in acetonitrile. The column was pre-equilibrated with 3% mobile phase B followed by a linear increase of 3–50% mobile phase B in 50 min. Mass spectra were acquired in a data-dependent mode utilizing a single MS survey scan (*m*/*z* 350–1,500) with a resolution of 60,000 in the Orbitrap, and MS/MS scans of the 20 most intense precursor ions in the linear trap quadrupole.

### Database Search

MaxQuant software (version 1.5.2.8) was used for peptide identification. A customized database featuring reviewed and non-redundant Uniprot human proteins (accessed March 2017) combined with 200 highly enriched bovine proteins found in FCS (21) was used for peptide identification. No enzyme specificity was used for the search, and a tolerance of 10 ppm was allowed for the main ion search and 0.35 Da for the MSMS identification. The “match between runs” feature was enabled. The FDR was set at 0.01 (1%). Reverse IDs and known contaminants like keratins were filtered before further data analysis.

### MS Data Use and Analysis

The analysis of the HLA-DQ2.2, 2.5, and 7.5 immunopeptidomes is based on the data reported by Bergseng et al. (13). Data (peptides.txt, Evidence.txt, and Protein.txt) files were retrieved from the repository: PRIDE/ProteomeXchange: http://www.ebi.ac.uk/pride/archive/projects/PXD001205.

The mass spectromeric datasets analyzed in this study are available in the PRIDE Archive (Project PXD008775, available at https://www.ebi.ac.uk/pride/archive/projects/PXD008775). The PLAtEAU algorithm is available as a Python script at https://github.com/e-morrison/plateau.

### Label-Free Quantification

Two different approaches were used to quantify the peptides eluted from the HLA-DR3 molecules of each cell line. On the one hand MaxQuant provides quantitative information at the peptide level assigning the area under the curve. The program uses acquisition features such as mass width, retention time, and MS1 ion intensity, then calculates the value using a 3-D approach yielding an ion peak volume. Default settings require a minimum of two MS counts. On the other hand, peptide landscape antigenic epitope alignment utility (PLAtEAU) utilizes the MS1 ion intensities of all peptides bearing the epitope under consideration. In both cases the values determined for each peak (peptide) or epitope (integrating several peptides) were normalized to the total sum of peak volumes or MS1 ion intensity scans in the same sample. Such an approach facilitates relative, direct comparison between samples.

### Other Bioinformatics Tools

Peptide-binding affinity predictions were retrieved from the NetPanMHCII server (22) using the amino acid sequences specified in each case. Gene Ontology analysis was done based on the Uniprot IDs loaded directly on the Panther server. GO-Slim cellular component enrichment terms were retrieved from the output (23). When no GO-Slim cellular component was given to an entry, the terms assigned in Uniprot were manually annotated. Seqlogos of the binding cores were generated using the Weblogo3 online tool (24) using as input the 9mers retrieved as most likely binding registers from the NetPanMHCII binding prediction.

### Statistical Analysis

GraphPad Prism 7.0 software (GraphPad Software, San Diego CA, USA) was used for statistical analysis of quantitative Western blot. Variance was calculated with the two-way ANOVA method. The null hypothesis was rejected when the *P*-value was lower than 0.05.

Perseus software (25) was mainly used to analyze the MS data. Either peptides (peak volumes) or epitopes (% intensity from the total ion current) determined by PLAtEAU were loaded as matrices. All data were log2-transformed and missing values were imputed as the minimum observed value (26). For heat map representation, columns were hierarchically clustered with “average” as the agglomeration method and “Pearson correlation” as the distance matrix. Rows were ordered by hierarchical clustering using “average” as agglomeration method and “Euclidean” as distance matrix.

To evaluate whether there were quantitative differences between the peptides or epitopes eluted from each DM condition all measurements from each condition were grouped and used to define the mean intensity value for each peptide or epitope. Using the software Perseus, *P*-values were calculated based on the observed intensities using a *t*-test, and setting the FDR to 0.01 and the value S0 to 0.2. For more details on the statistical testing applied see Ref. (27). These FDR and S0 cutoffs may be adjusted to be more or less strict, depending on the degree of confidence desired.

## Results

### Rationale, Design, and Features of PLAtEAU

Previous approaches to quantitatively defining the immunopeptidome displayed by MHCII molecules have mostly focused on individual peptides identified (12–14). Peptides differing in length by only one or several amino acids are frequently found as products of cathepsin-cleaved proteins and are treated as separate epitopes, even if they share a common binding motif. To date there is only one report in which quantitative information from sets of nested peptides have been analyzed by a proteomics approach. Since this report performed analysis of only 14 sets of nested peptides that were manually annotated (17) we aimed to expand the concept to large data sets. We thus developed an algorithm for grouping peptides into consensus epitopes based on nested peptides, representing the shared sequence (epitope) that is presented by individual peptides displayed on the cell surface; these epitopes can then be quantified using conventional label-free or isotopic quantification strategies.

**Figure 1:**
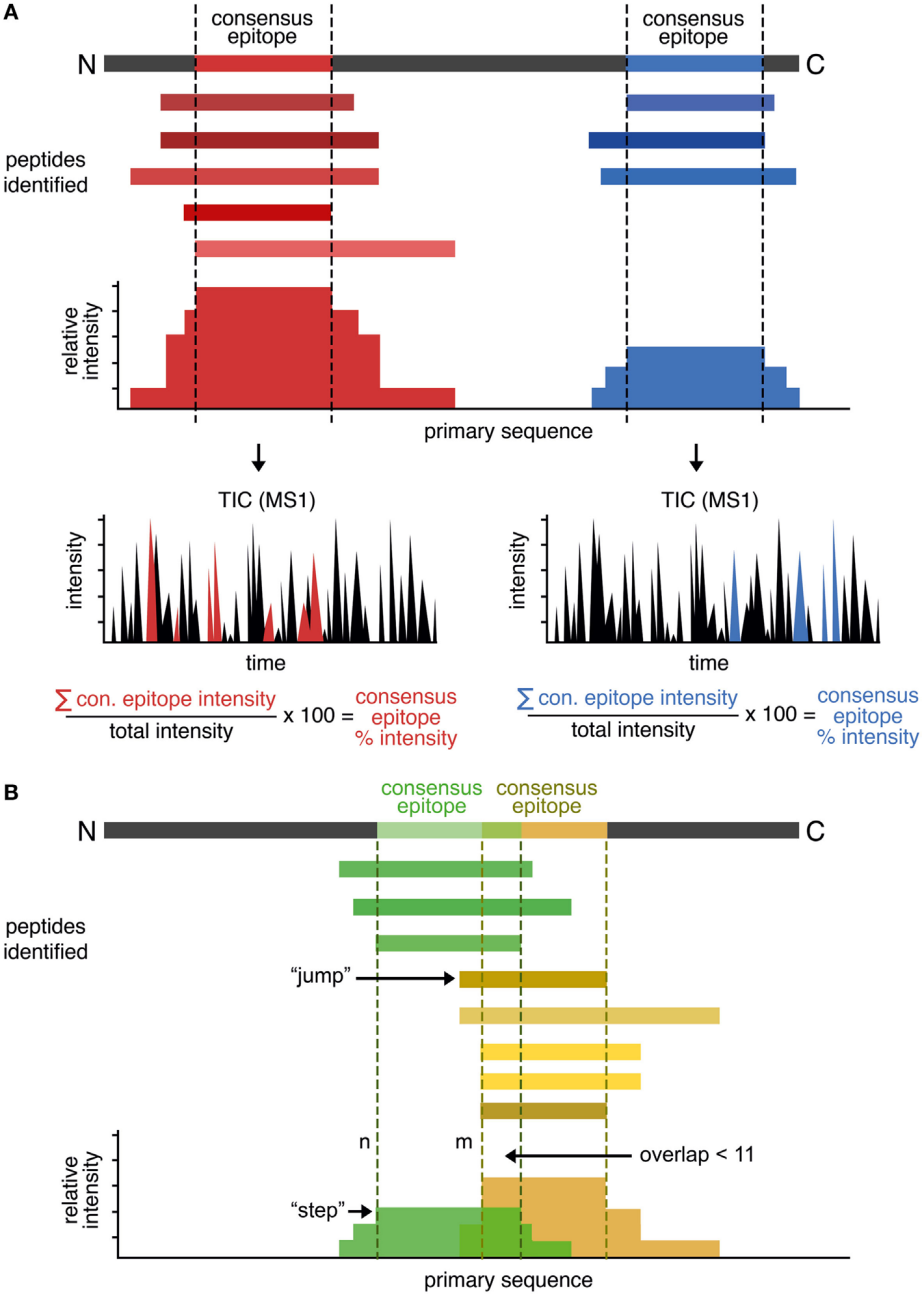
Rationale of the peptide landscape antigenic epitope alignment utility (PLAtEAU) algorithm. **(A)** The algorithm aligns each identified peptide to the primary sequence of the database-matched protein entry (parent protein). Series of nested peptides are grouped and aligned, and the total intensity is calculated at a per-residue level. The intensity “plateaus” define the consensus epitopes. The algorithm also retrieves the sum of all MS1 intensity values of each specific LC–MS/MS run, yielding the relative percentage intensity of the consensus epitope. **(B)** To detect frame-shifted epitopes, peptides are aligned to the primary protein sequence and ordered by the N-terminal residue position (*n*). The distance to the next peptide’s N-terminus (*n* + *m*) is analyzed, usually leading to “steps” in the plateau. “*Jumps*” of five or more residues to the next peptide (*m* ≥ *n* + 5) will define a second, frame-shifted epitope when the next peptide does not overlap with the directly preceding peptide (*n*) by 11 residues or more. Peptides are then segregated into two groups: those with N-terminal positions before or at *n*, and those with N-terminal positions at or after *n* + *m*. These segregated peptide pools are then used to generate two new PLAtEAU distributions, as in panel **(A)**.

Our approach is summarized in Figure 1A. At first peptides eluted from HLA-DR and identified by LC–MS/MS are aligned to the *in silico* primary sequence of the appropriate parent protein. We then calculate the total intensity value on a *per-*residue basis by summation of the intensities of all peptides that contain this particular site. This gives rise to an intensity “landscape,” with “plateaus” representing the shared sequence among the identified peptides; we take these “plateaus” to be the “consensus epitope” of the protein. Consequently, a protein containing multiple consensus epitopes will be identified and distinguished by several plateaus. Each of the shared epitopes are defined by a minimum length of 11 amino acids, as we reasoned that these constraints will favor the selection of the core binding epitope (9 residues) plus at least one residue on each side of the binding epitope. To account for the additional influence of residues in longer sequences we calculate the average number of residues as N- and C-terminal overhangs.

In some cases, nested peptide sets could overlap due to the presence of multiple binding registers separated by a small number of residues. Register shifting refers to the potential ability of peptides to bind MHCII molecules utilizing different anchor residues [e.g., CLIP1, CLIP2, and CLIP3 for HLA-DQ, as observed previously (13)]. This effect results in misleading “consensus epitopes” that do not take into account the overlapping populations of nested peptides. To overcome this, the PLAtEAU script was modified to capture defining features of these overlapping sets and to deconvolute register-shifted epitopes overlapping by five or more residues. First, all peptides were aligned to the primary protein sequence and ordered by the N-terminal residue position (*n*) (Figure 1B). Then, the distance to the next peptide’s N-terminus (*n* to *m*) was calculated. Due to the nature of the mixture of exo- and endoproteases in the endosome, this distance typically is one to two residues in length and gives rise to distinct steps between “plateaus” (*m* = *n* + 1 or *n* + 2); if a “step” of 5 or more residues to the next peptide (*m* ≥ *n* + 5) is found, it is considered a “jump,” and when the next peptide does not overlap with the directly preceding peptide (*n*) by 11 residues or more, this is considered to constitute a second epitope that binds in a shifted register, as seen in CLIP1, CLIP2, and CLIP3, mentioned earlier. When such a pattern is identified, the peptides are segregated into two groups: those with N-terminal positions before or at *n*, and those with N-terminal positions at or after *n* + *m*. These segregated peptide pools are then used to generate two new PLAtEAU distributions, as described earlier.

We further implemented a method of calculating a label-free relative quantification of these consensus epitopes similar to that described before (13, 17). Essentially, we calculate the sum of all MS1 intensity values of the peptides used to define a given consensus epitope and, by dividing this number by the sum of all MS1 intensity values in the specific LC–MS/MS measurement, we obtain the relative percent intensity of the consensus epitope. This value corresponds to the degree that a given consensus epitope is represented in one sample and can be directly compared across different conditions (see subsequent sections).

### Benchmarking PLAtEAU With the Previously Reported HLA-DQ Immunopeptidome (13)

**Figure 2:**
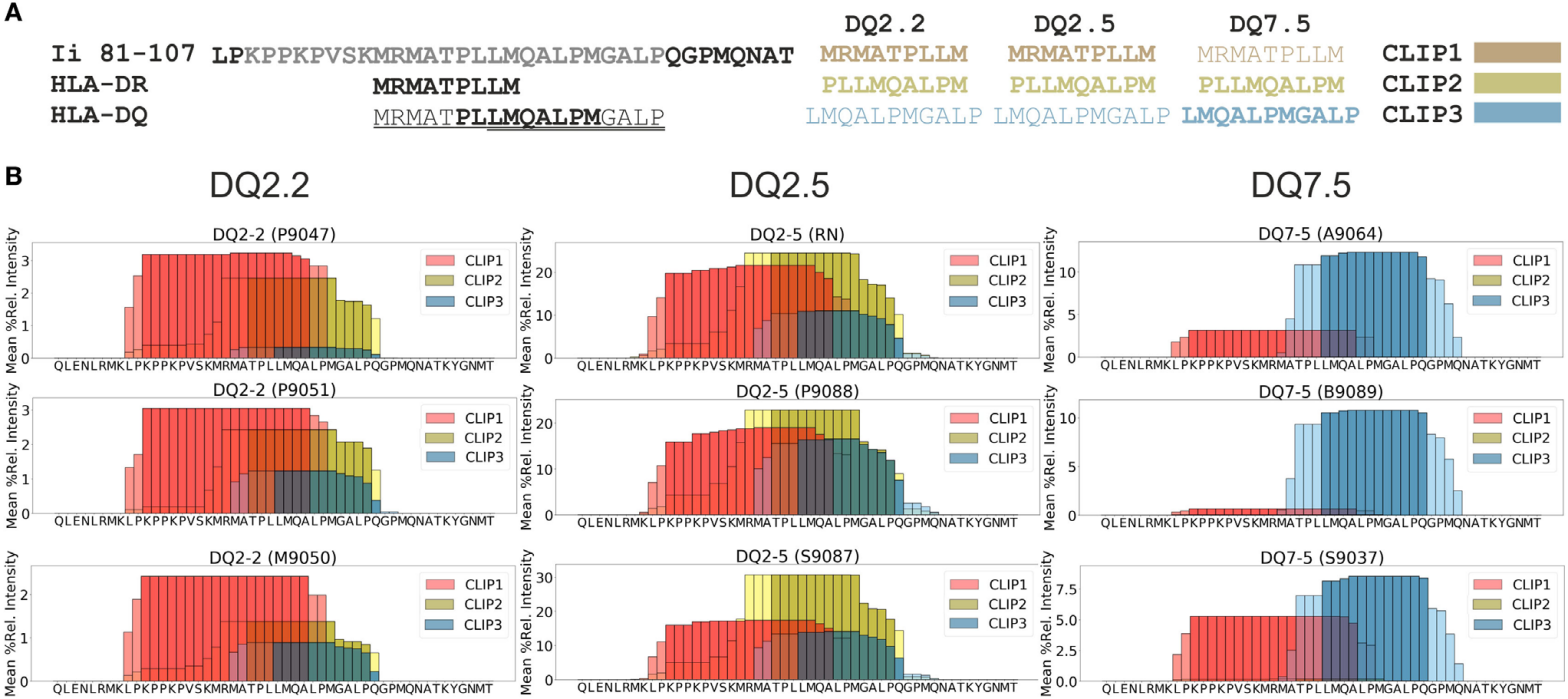
Analysis of the dataset published in Ref. (13) with the peptide landscape antigenic epitope alignment utility algorithm. **(A)** Class II invariant chain peptide (CLIP)-derived peptides binding to major histocompatibility complex of class II molecules. As a point of comparison, HLA-DR molecules bind mostly the peptide spanning residues 91–99, while HLA-DQ molecules bind three different overlapping peptides in the region 91–108. Each of the three CLIP-derived epitopes described binding to HLA-DQ molecules is shown, with the corresponding color legend shown in panel **(B)**. Different HLA-DQ allotypes bind preferentially the epitope(s) shown in bold letters. **(B)** “Plateaus” can be identified based on spectral counting and/or relative intensities for CLIP-derived epitopes from CD74 (Uniprot accession code P04233). In this particular case, the mean relative intensities of each amino acid are shown (see Table S2 in Supplementary Material). Darker colors represent the core epitopes, and the light colors represent extended areas covered by peptides. The Uniprot entry code of each cell line used in the original studies (in brackets) is also provided above each “plateau” of the various conditions analyzed in Ref. (13).

We were interested in testing our criteria for peptide identification and quantification as well as the performance of PLAtEAU on a curated dataset. To this aim we chose a recent study of the HLA-DQ immunopeptidome (13), performed in a similar experimental approach as our own. The dataset was retrieved from the PRIDE/ProteomeXchange repository (see Materials and Methods). The file containing all peptides was processed by the PLAtEAU algorithm, resulting the relative consensus epitope intensities (Table S1 in Supplementary Material). Our analysis retrieved approximately 650 epitopes in the whole dataset, with an average overhang length of the N- and C-terminal extensions less than 1.25 residues (Figures S1 and S2 in Supplementary Material). As a paradigmatic example of a nested peptide analysis, we chose peptides derived from CLIP, which were shown to bind to DQ molecules in at least three different binding registers (CLIP1, CLIP2, and CLIP3), all of which are included in the amino acid sequence spanning residues 81–107 of the invariant chain (Ii) (Figure 2A) (13). DQ2.2 and DQ2.5 molecules bind CLIP1 and CLIP2 (28, 29), and CLIP3 was hypothesized as an additional binding register for DQ7.5 (13). PLAtEAU is able to deconvolute the series of nested peptides and identify the binding consensus epitopes described for DQ2.2 and DQ2.5. As reported previously (13), CLIP3 is found here to be the preferential epitope bound to DQ7.5, and the most likely binding register would include the amino acid sequence LMQALPMGALP (Figure 2B; Table S2 in Supplementary Material). Finally, quantification of the sum of all CLIP-derived peptides by PLAtEAU yields a similar result as previously described, with average relative intensities of 59% (our study) vs. 52% (13) for DQ2.5, 5.8 vs. 5.7% for DQ2.2 and 13.7 vs. 11.8% for DQ7.5, respectively (Table S2 in Supplementary Material).

### Characterization of T2-DR3 Cell Lines Stably Expressing DM Allotypes

**Figure 3:**
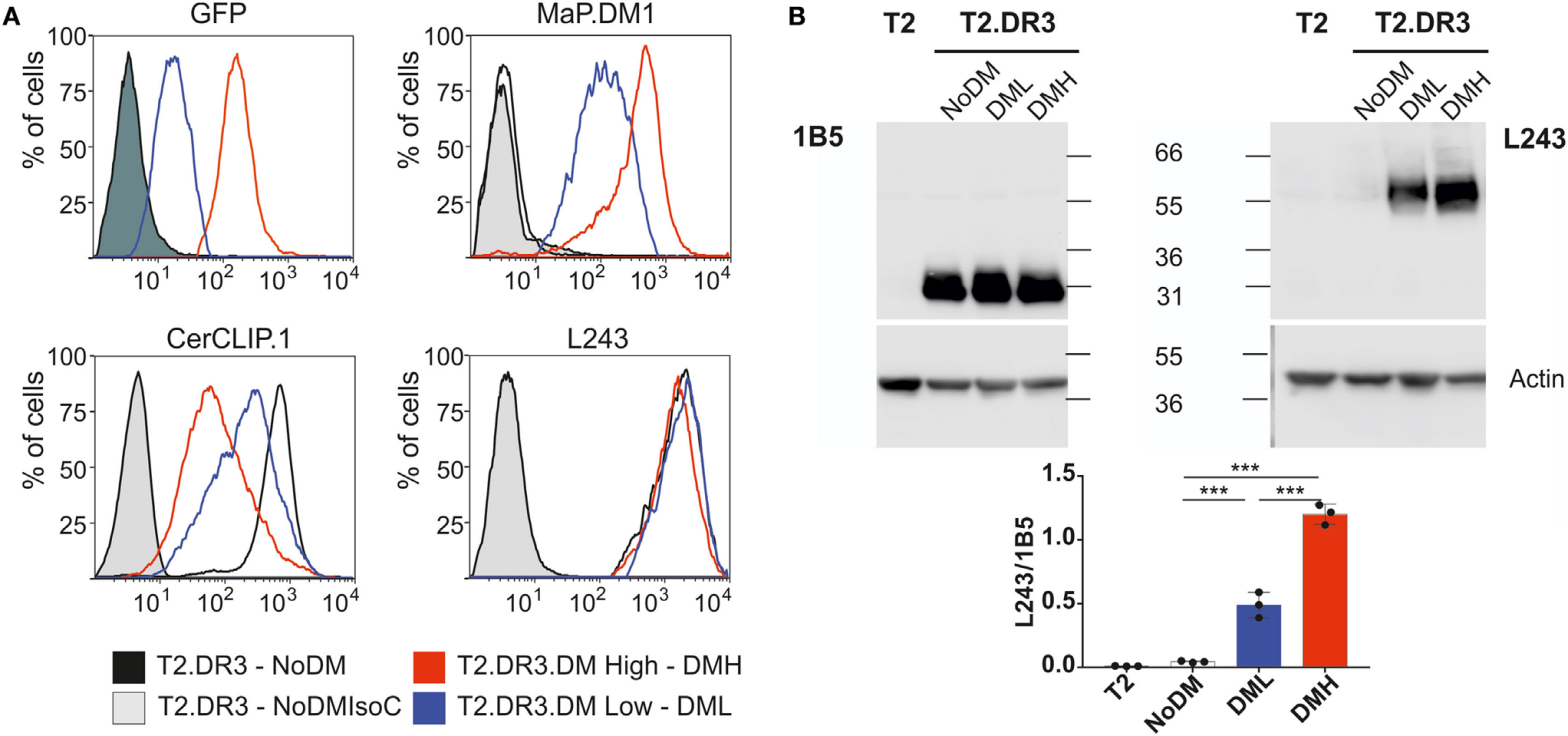
Characterization of cell lines expressing different levels of HLA-DM. **(A)** Representative flow cytometry histograms of the cell lines used in this study. The T2, T2-DR3 cell line lacking DM, and individual clones of T2-DR3 cell lines transduced with HLA-DM were grown in standard cell culture conditions. Cells in the mid-logarithmic exponential growth phase were stained for the fluorescent proteins indicated in each case. Shaded histograms show either the non-transduced cell line (upper left, dark) or isotype controls (light gray). Stainings of the T2 cell line essentially overlap with the non-transduced cell lines (GFP) and the isotype controls (antibody stains, and therefore are not shown). **(B)** SDS-stable dimer assay of the cell lines shown in panel **(A)** by Western blot (upper panel) and quantification of the ratio stable dimer vs. DRB under conditions of no, low or high DM expression. Post nuclear cell lysates are divided and resuspended in loading buffer and either boiled (left) or kept at room temperature for 5 min (right). Samples (20 µg) are loaded and resolved in a 10% SDS-PAGE gel. The gel was subsequently blotted and probed either for detection of DRB (left, TAL 1B5 Abcam AB20181) or stably folded DR heterodimers (right, L243 produced in house), and the signal was detected in both cases using a goat-anti-mouse-HRP (Santa Cruz) and a commercial luminol-based reagent. In both cases, β-actin detection was used as loading control using a mouse-anti-beta-actin-HRP antibody (AC-15 Abcam AB49900). The Western blot shown is a representative example of one of three independent experiments. The quantification is the result of *n* = 3 independent experiments measured twice each (ANOVA test: *P* < 0.0001).

The T2-DR3 cell line was transduced with lentiviral particles encoding for HLA-DM. The construct design allows the detection of GFP as a surrogate expression marker for DM, since both proteins are expressed from the same transcript. Cells were initially sorted based on the expression of GFP (Figure 3A, upper left panel), and subsequently single cell clones were isolated. We selected two clones based on GFP expression levels, one high and one low. HLA-DM expression levels were independently determined by intracellular staining of HLA-DM. Subsequent flow cytometry analysis allowed us to quantify the CLIP surface display, as well as the HLA-DR expression levels. Staining for CLIP reveals that 95% of DM-negative cells show a strong signal of surface MHCII–CLIP complexes, and that upon HLA-DM expression this signal is considerably reduced (Figure 3A, upper right panel), while HLA-DR expression remains almost unaltered (Figure 3A, lower right panel). These results confirm that the expressed HLA-DM molecules yield functional heterodimers, and that expression levels of GFP are paralleled by the expression levels of HLA-DM. Moreover, the relative amount of CLIP displaced from DR3 molecules is inversely proportional to the HLA-DM expression level and is significantly different between the two clones tested (Table 1).

**Table 1:** Flow cytometry of relevant epitopes of the cell lines used in this study.

	No DM	Low DM	High DM
Class II invariant chain peptide	597 ± 14	244 ± 9	119 ± 20
GFP	4.5 ± 1.8	40.5 ± 6	109 ± 18
HLA-DM	24.2 ± 4	127.2 ± 22	333.1 ± 23
HLA-DR	1,105 ± 98	1,382 ± 121	1,526 ± 135

*gMFI values ± SD of *n* = 3 independent experiments determined in duplicates. Values for T2 cells are in the range of those of the non-transduced cell lines (GFP) or the isotype controls (antibody stains)*.

HLA-DM function favors the selection of pMHCII complexes with high kinetic stability, a feature that is correlated to the presence of SDS-stable dimers in PAGE analysis. In the particular case of T2-DR3, it has been shown that such complexes are only formed in the presence of HLA-DM (30). Thus, the expression levels of HLA-DM are directly proportional to the formation of SDS-stable dimers in I-A^b^ MHCII molecules (31). We assessed the presence of SDS-stable dimers for the clones with no, low, or high HLA-DM expression levels and investigated whether HLA-DM expression affects the formation of the SDS-stable dimers. First, the amount of HLA-DR3 in each cell line was determined (measured as HLA-DR3 beta chain signal) (Figure 3B left). Second, the amounts of HLA-DR present in the SDS-stable dimeric conformation were analyzed (Figure 3B right). Western blot quantification using ratios of SDS-stable dimers (detected by L243) and of DRA (using 1B5) then allowed us to confirm that the extent of SDS-stable dimers formed under these conditions depended on the relative expression levels of HLA-DM (Figure 3B low). In summary, our results confirm that the CLIP epitope is replaced by high-affinity peptides, and that this display is dependent on the expression levels of HLA-DM.

### PLAtEAU Reduces the Complexity of Immunopeptidome Datasets

**Figure 4:**
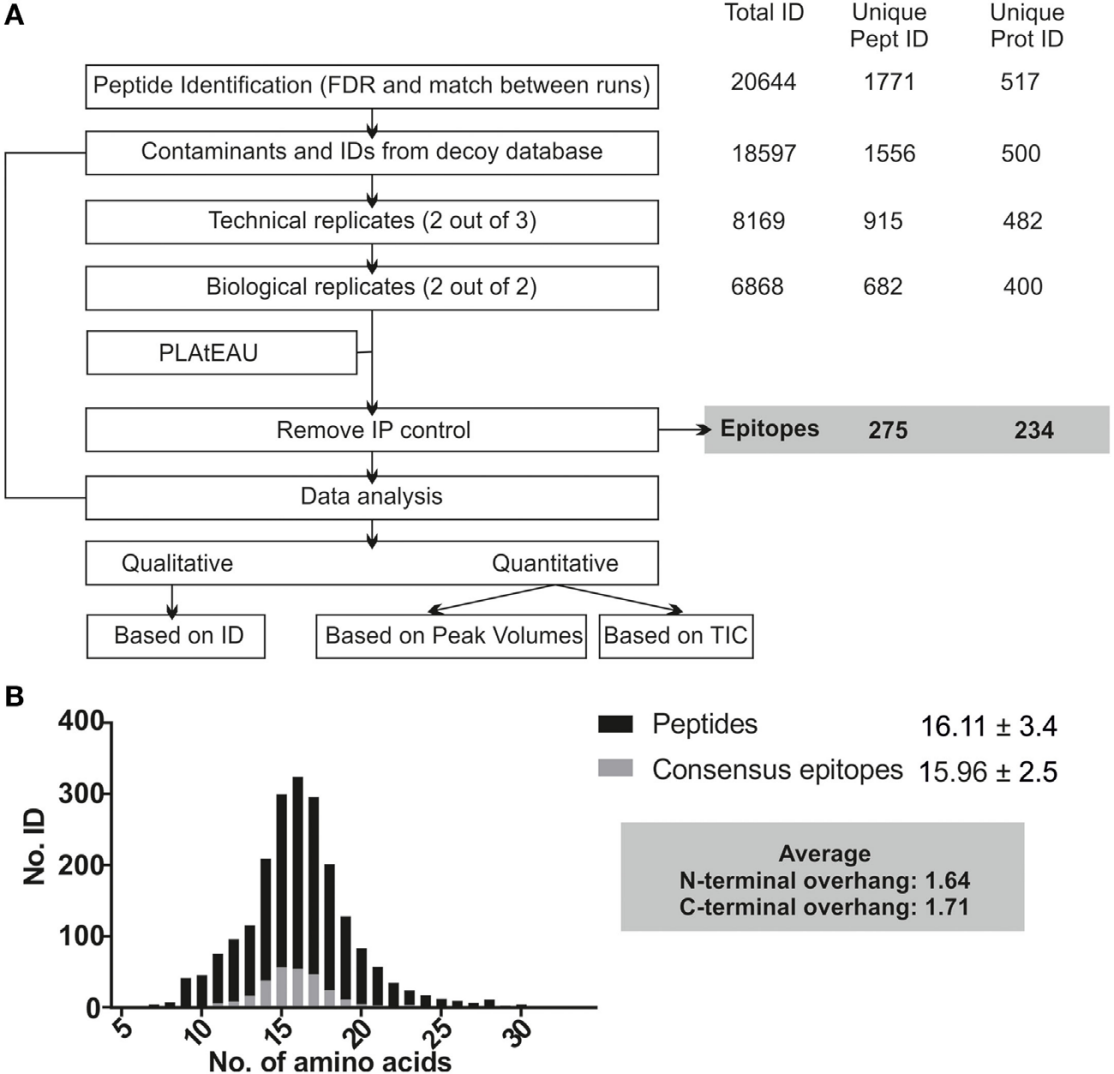
Experimental summary of the analysis of the DM-dependent immunopeptidome associated with HLA-DR3. Cell lines were grown in two independent experiments and originally divided in three pellets that were processed independently for each biological replicate. **(A)** Scheme of the streamlined immunopeptidome analysis utilizing peptide landscape antigenic epitope alignment utility (PLAtEAU). Peptide identification with MaxQuant includes an FDR of 0.01 and allows identification of peptides by the “match between runs” option across the entire set of replicates (time window of 0.5 min with an alignment time window of 20 min). Known contaminants (identified by an internal database in MaxQuant) and identifications from the decoy database were first removed. The resulting dataset is used for comparison with our approach. For PLAtEAU analysis, we applied two extra filters, one for technical and another one for biological replicates. This dataset was analyzed with the PLAtEAU script, and subsequently we removed the epitopes detected in the immunoprecipitation control samples. The number of identifications, peptides, and protein sources is shown on the right side. The PLAtEAU script yielded 275 core epitopes from 234 protein sources. This dataset was used for qualitative and quantitative analysis. **(B)** Size distribution and average length values based on the number of identifications at the peptide (black) and the consensus epitope level (gray).

The MHCII immunopeptidome associated with the cell lines described earlier was isolated after whole cell lysis, immunoaffinity purification, and acid elution of the peptides bound to MHCII molecules. After LC-MS analysis, raw files were analyzed using the MaxQuant software with the parameters described in Section “Materials and Methods,” applying a FDR of 0.01 and the “match between runs” feature. As depicted in Figure 4A, only peptides found in both biological replicates and at least two out of three technical replicates were considered for the PLAtEAU analysis. A total of 20,644 peptides from 1,771 unique peptides or 517 unique proteins were identified across all of the different samples and conditions, including potential contaminants and identifications from the decoy database. Removal of such contaminants reduced the numbers by around 10%. Filtering for the technical and biological replication criteria decreased these values further, to around one-third of the original peptide IDs. Processing the pool of peptides resulting from these filters with the PLAtEAU algorithm yields a dataset consisting of 275 total consensus epitopes from 234 total protein sources (Figure 4A; Table S3 in Supplementary Material).

The PLAtEAU peptide alignment strategy does not rely on peptide-binding motifs, nor does it restrict the peptide length beyond the minimum of 11 and the maximum of 30 amino acids. Peptide and consensus epitope size distribution is very similar (Figure 4B). Moreover, the average length of the C-terminus extending beyond the consensus epitope is 1.71 residues, while it is 1.64 residues in the N-terminal direction. Apparently, there is no bias for extensions at the peptide level on either terminus. It is worth noting that we could detect a large set of peptides that must be considered as background binders during the immunoaffinity purification, as they are enriched in the control condition using cell lysates of T2 cells not expressing any HLA-DR molecule (Figure 4A; Figure S3A in Supplementary Material).

We further evaluated the effect the biological replicate and immunoprecipitation (IP) controls had on the final curated immu-nopeptidome analyzed. Venn diagrams showing the overlap between biological replicates only including IDs found in two out of three technical replicates are shown in Figure S3 in Supplementary Material. Overall, the number of peptide IDs found for each sample increases upon HLA-DM expression, and, similarly, the overlap between biological replicates increases from 60% to more than 80% upon HLA-DM expression (Figures S3A,B in Supplementary Material). After background removal, samples lacking DM expression exhibit a considerable degree of discrepancy (60% overlap) between biological replicates, in contrast to those expressing DM (approx. 80% at the peptide level and >90% at the epitope level). These results suggest a generally more unstable and variable immunopeptidome displayed on the surface of cell lines not expressing DM.

### Distinct Abundances of Peptides Within the HLA-DR3 Immunopeptidome Determined by LFQ of PLAtEAU-Derived Epitopes

**Figure 5:**
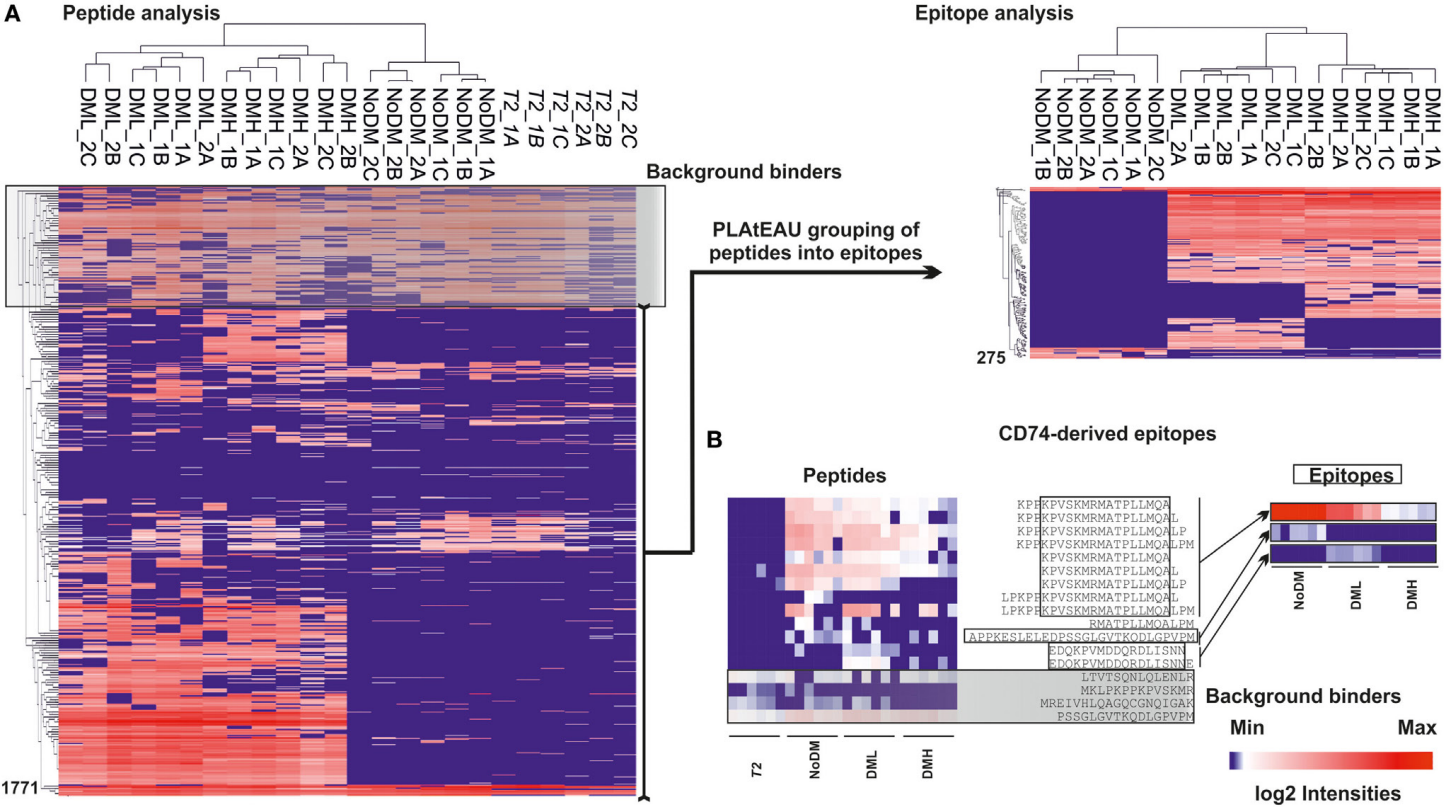
Heat map representation of the T2-DR3 immunopeptidome using MaxQuant peak volumes and the relative intensities retrieved with peptide landscape antigenic epitope alignment utility (PLAtEAU). **(A)** Left side shows the hierarchical clustering based on LFQ relative intensities determined by MaxQuant for each peptide. DML stands for low DM expression and DMH for high DM expression. NoDM samples do not express HLA-DM, and T2 indicates samples obtained from T2 cell lines not expressing HLA-DR3. The black box shaded in gray (background binders) on the left hand heatmap indicates peptides identified in the immunoprecipitation control samples, which were subsequently regarded as false positives. Those peptides are therefore removed from further analysis and do not appear in the epitope heatmap. On the right side, non-background peptides obtained after removal of background binders and grouping into epitopes by the PLAtEAU algorithm are also hierarchically clustered. Numbers on the left sides of the heat maps indicate the counts of the peptides or epitopes. **(B)** Example of performance of the experimental approach for CD74-derived peptides and their grouping into epitopes shown as heat maps before (left) and after (right) processing the dataset as described earlier. The peptide sequences are shown in one-letter amino acid code. Open boxes indicate the identified consensus epitope, and the gray box shows the background binders identified for peptides derived from this protein. The log2 intensity barscale shown in the left corner is indicative of the color code of al heat maps.

Another key feature of the PLAtEAU algorithm is that it allows for LFQ of the grouped consensus epitopes. Despite the considerable increase in available datasets from MHCII immunopeptidome studies in recent years, only a handful have made use of LFQ strategies to quantify the extent of presentation of relevant epitopes. The PLAtEAU algorithm offers an improvement compared with previously described approaches by aligning quantified nested peptides and identifying the consensus binding epitopes, which can then be quantified themselves across various samples and conditions. The resulting LFQ values for individual peptides obtained from the MaxQuant output files were plotted as a heatmap (Figure 5A left), as reported previously (13). Removal of background binders and grouping of peptides into consensus epitopes with PLAtEAU (Figure 5A left) yields a reduced heatmap consisting of 275 epitopes. The performance of the strategy is illustrated by the CLIP peptides derived from CD74 (invariant chain) (Figure 5B). In this case, several peptides are found in the samples generated from the T2 cells lines used as IP control and are therefore considered as false positives. In addition, grouping of sets of nested peptides into consensus epitopes (e.g., KPVSKMRMATPLLMQA) makes apparent differences in the relative amount of peptides found for each condition.

**Figure 6:**
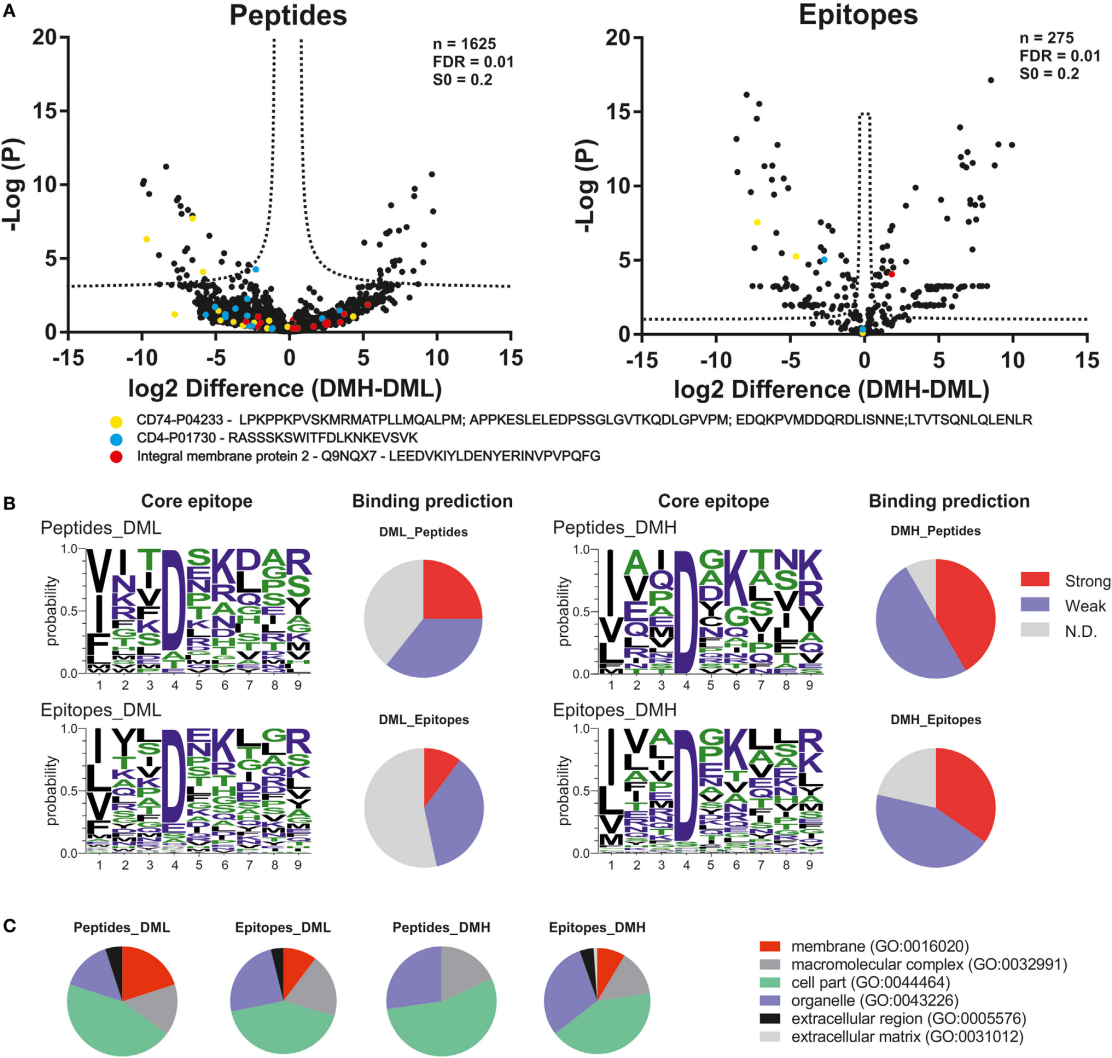
Quantitative differences in the immunopeptidome of T2-DR3 cell lines depending on the DM expressing levels. **(A)** Volcano plots showing the log2-fold change of intensity of each peptide quantified by MaxQuant or each epitope quantified by peptide landscape antigenic epitope alignment utility vs. the −log(P). In both cases, an FDR of 0.01 and an S0 of 0.2 as correction factor for differences in the means were used. The resulting intervals of confidence are highlighted by dashed lines shown in each graph. Peptides and epitopes from three representative protein entries are shown as colored filled circles: yellow: CD74; blue: CD4; red: integral protein membrane 2. **(B)** NetPanMHCII was used to predict the core epitope (Seqlogo representation) of the peptides and epitopes enriched for each condition and their relative binding affinity (pie charts). The relative affinities are provided as stated directly from the NetPanMHCII analysis as Strong for strong binders, Weak for weak binders, and N.D. for those cases in which a binding core was not determined. **(C)** Cellular component GO analysis of the peptides and epitopes enriched for each condition.

We then analyzed the impact of DM expression levels on the immunopeptidome and the potentially beneficial consequences of grouping peptides into consensus epitopes for quantitative purposes. The fold change in intensities between low DM expression (DML) and high DM expression (DMH) samples was plotted vs. the *t*-test *P*-value for each peptide or each epitope (Figure 6A left and right, respectively). It is worth noting that we took into consideration the same FDR and the artificial between-groups variance [see Ref. (27) for details]. The confidence interval for each dataset is shown for each case as a dotted line. At first sight the distribution of data points clearly changes upon peptide grouping into epitopes. Thus, the volcano plot for the peptide dataset (Figure 6A left) shows most of the data points accumulated close to the *x*-axis while there is a more sparse distribution in case of the epitopes retrieved after using PLAtEAU (Figure 6A right). Three specific protein entries with either peptides or epitopes highly abundant for each condition (Table 2) illustrate that when comparing abundances at the peptide level, most of the data points lie outside of the interval of confidence, while grouping them into consensus epitopes yields more high confidence hits (Table 3; Figure 6A). In summary, only 52 data points lie within the confidence interval in the case of the analysis of peptides, while 186 can be considered as enriched when analyzing the consensus epitopes (see Tables S4 and S5 in Supplementary Material).

**Table 2:** Ten most intense peptides or epitopes found by MaxQuant and peptide landscape antigenic epitope alignment utility (PLAtEAU).

DR3 MaxQuant	DR3 PLAtEAU	DML MaxQuant	DML PLAtEAU	DMH MaxQuant	DMH PLAtEAU
KPPKPVSKMRMATPLLMQALP (P04233; 21.40) CD74—ORGANELLE; MEMBRANE	KPVSKMRMATPLLMQA (P04233; 94.79) CD74—ORGANELLE; MEMBRANE	LPKPPKPVSKMRMATPLLMQALPM (P04233; 6.962) CD74—ORGANELLE; MEMBRANE	KSWITFDLKNKE* (P01730; 17.42) CD4—CELL PART; MEMBRANE	EDVKIYLDENYERINVPVP (Q9NQX7; 8.270) Integral membrane protein 2— ORGANELLE; EXTRACELL; CELL PART	KIYLDENYERIN (Q9NQX7; 15.93) Integral membrane protein 2—ORGANELLE; EXTRACELL; CELL PART

KPPKPVSKMRMATPLLMQALPM (P04233; 13.27) CD74—ORGANELLE; MEMBRANE	VDDTQFVRFDSDAASQ (P01891; 1.251) HLA-A—MEMBRANE	KSWITFDLKNKEVSVK (P01730; 6.246) CD4—CELL PART; MEMBRANE	KPVSKMRMATPLLMQA (P04233; 12.14) CD74—ORGANELLE; MEMBRANE	FQNTIIFDNKAHSGKI (Q8IZK6, 3.392) Mucolipin—ORGANELLE; MEMBRANE; CELL PART	KSWITFDLKNKE* (P01730; 8.021) CD4—CELL PART; MEMBRANE

KPVSKMRMATPLLMQAL (P04233; 11.45) CD74—ORGANELLE; MEMBRANE	AAVVVPSGQEQRYT (P01892; 0.837) HLA-A—MEMBRANE	SKSWITFDLKNKEVSVK (P01730; 2.550) CD4—CELL PART; MEMBRANE	ITSIVKDSSAARN (O00560; 7.304) Syntenin-1—CELL PART	DGVIKVFNDMKVRKSSTPE (P23528; 2.433) Cofilin 1—ORGANELLE; CELL PART	ITSIVKDSSAARN (O00560; 7.743) Syntenin-1—CELL PART

KPPKPVSKMRMATPLLMQAL (P04233; 6.866) CD74—ORGANELLE; MEMBRANE	PRKIEEIKDFLLTAR (P63173; 0.498) 60S L38 ribosomal protein—ORGANELLE; MACR. COMPLEX; CELL PART	VPAVVIDMSGLREKDD (P13796; 2.394) Plastin 2—ORGANELLE; MACR. COMPLEX; CELL PART	KIYLDENYERIN (Q9NQX7, 5.933) Integral membrane protein 2—ORGANELLE; EXTRACELL; CELL PART	EDVKIYLDENYERIN (Q9NQX7; 2.381) Integral membrane protein 2—ORGANELLE; EXTRACELL; CELL PART	SVIIVDKNGRL (P02786; 5.118) Transferrin receptor protein 1—ORGANELLE; MEMBRANE

LPKPPKPVSKMRMATPLLMQALPM (P04233; 6.516) CD74—ORGANELLE; MEMBRANE	DDDIAALVVDNGSGMCKA (P60709, 0.313) Actin cytoplasmic—ORGANELLE; CELL PART	EDVKIYLDENYERIN (Q9NQX7; 2.308) Integral membrane protein 2—ORGANELLE; EXTRACELL; CELL PART	SVIIVDKNGRL (P02786; 5.533) Transferrin receptor protein 1—ORGANELLE; MEMBRANE	ITSIVKDSSAARNGLL (O00560; 2.207) Syntenin-1—CELL PART	IFDNKAHSGKI (Q8IZK6; 4.229) Mucolipin—ORGANELLE; MEMBRANE; CELL PART

SGKKLEDGPKFLK (P68104; 2.746) Elongation factor 1 A1—**NUCLEUS	ASASGAMAKHEQILVLD (Q9P0L0; 0.254) Vesicle-associated membrane protein-associated protein A—ORGANELLE; MEMBRANE	KSWITFDLKNKEVS (P01730; 2.283) CD4—CELL PART; MEMBRANE	VDDTQFVRFDSDAASQ (P01891; 4.225) HLA-A—MEMBRANE	KSWITFDLKNKEVSVK (P01730; 2.024) CD4—CELL PART; MEMBRANE	VIKVFNDMKVRKSSTPE (P23528; 3.917) Cofilin 1—ORGANELLE; CELL PART

KPVSKMRMATPLLMQALP (P04233; 2.331) CD74—ORGANELLE; MEMBRANE	APGKGILAADESTGSIA (P04075; 0.246) Fructose bisphosphate aldolase A—** CELL PART; EXTRACELL	QLLSFVRDLNQYRADIK (P02786; 2.223) Transferrin receptor protein 1—ORGANELLE; MEMBRANE	GPPKLDIRKEEKQIMID (P15260; 3.458) Interferon gamma receptor 1—MEMBRANE	EDVKIYLDENYERINVPV (Q9NQX7, 1.889) Integral membrane protein 2—ORGANELLE; EXTRACELL; CELL PART	DPKRTIAQDYG (Q06830; 3.023) Peroxiredoxin 1 CELL PART

KPPKPVSKMRMATPLLMQA (P04233; 2.099) CD74—ORGANELLE; MEMBRANE	DRNTQIFKTNTQTYREN (P18464; 0.197) HLA-B—MEMBRANE	ITSIVKDSSAARNGL (O00560; 1.953) Syntenin-1—CELL PART	IFDNKAHSGKI (Q8IZK6; 2.501) Mucolipin—ORGANELLE; MEMBRANE; CELL PART	TPILVDGKDVMPEVN (P06744; 1.530) Glucose-6-phosphate isomerase—**s MEMBRANE, EXTRACELL; CELL PART	IRTIELDGKTIKL (P62820; 2.277) Ras-related protein Rab1A—ORGANELLE; CELL PART

PYVPVHFD (P07998; 1.833) Pancreatic RNASE1—**EXTRACELL	ALTVPELTQQMFDAK (P04350; 0.193) Tubulin B 4A—ORGANELLE; CELL PART	FQNTIIFDNKAHSGKI (Q8IZK6—ORGANELLE; MEMBRANE; CELL PART; 1.728) Mucolipin—lysosome, endosome	EPSRGINPDEAVAYG (P11021; 1.774) 78 kDa glucose-regulated protein—ORGANELLE; MACR. COMPLEX	KSWITFDLKNKEVS (P01730; 1.439) CD4—CELL PART; MEMBRANE	LLSFVRDLNQYRADIK (P02786; 2.120) Transferrin receptor protein 1—ORGANELLE; MEMBRANE

IGQGYLIKDGKLIKNNASTDYDLSDK (P39023; 1.565) 60S L ribosome—ORGANELLE; MACR. COMPLEX; CELL PART	VEVSVKSDDKHMHDHNH (Q9ULF5, 0.172) Zinc Transporter ZIP10—MEMBRANE	ITSIVKDSSAARNGLL (O00560; 1.702) Syntenin-1—CELL PART	VIKVFNDMKVRKSSTPE (P23528; 1.342) Cofilin 1—ORGANELLE; CELL PART	DPKRTIAQDYGVLKADEG (Q06830; 1.387) Peroxiredoxin 1—**EXTRACELL; CELL PART	KSINPDEAVAYG (P0DMV8; 1.959) Heat-shock 70 kDa protein 1A—ORGANELLE; MACR. COMPLEX; EXTRACELL; CELL PART

Classification is based on values obtained after normalization. The Uniprot accession code for the most likely protein source is indicated and the relative abundance of the peptide or epitope expressed as percent is shown within brackets. Asterisks (*) indicates that the epitope arises from the overlap of two potential register-shifted epitopes and indicates the position (N- or C-terminal) of the “jump” (see Figure 1), i.e., the area where the epitopes begin to overlap. The subcellular localization based on GO analysis is also provided and where stated as (**) manual annotation based on GO terms indicated in Uniprot entry was used.

**Table 3:** Enrichment behavior of peptides and epitopes of three abundant protein entries.

MaxQuant-peptides				Uniprot accession	PLAtEAU-epitopes			
Sig.	−log(*P*)	Diff.	Sequence		Sig.	−log(*P*)	Diff.	Sequence
	0.608	−1.124	KSWITFDLKNKE	P01730 CD4	+	4.042	−1.134	KSWITFDLKNKE*
	1.096	−2.279	KSWITFDLKNKEV					
	2.360	−1.443	KSWITFDLKNKEVS					
	1.259	−1.705	KSWITFDLKNKEVSV					
	2.162	−2.503	KSWITFDLKNKEVSVK					
	0.170	−1.086	RASSSKSWITFDLKNKEVSVK					
	0.414	−1.178	SKSWITFDLKNKE					
	0.070	−0.675	SKSWITFDLKNKEV					
	2.935	−1.241	SKSWITFDLKNKEVS					
	0.825	−2.376	SKSWITFDLKNKEVSV					
	1.420	−2.279	SKSWITFDLKNKEVSVK					
	0.182	0.729	SSKSWITFDLKNKE					
	0.005	−0.038	SSKSWITFDLKNKEV					
	1.897	−1.375	SSKSWITFDLKNKEVS					
	0.906	−3.922	SSKSWITFDLKNKEVSVK					
	0.495	1.496	SVKRVTQDPKLQMGKK		n.d.			
	0.160	−0.912	VKRVTQDPKLQMGKK			0.004	−0.022	*VKRVTQDPKLQMGKK
+	7.711	−6.558	EDQKPVMDDQRDLISNN	P04233 CD74	+	7.563	−7.223	EDQKPVMDDQRDLISNN
	1.684	−5.471	EDQKPVMDDQRDLISNNE					
	0.098	−0.605	APPKESLELEDPSSGLGVTKQDLGPVPM			0	0	APPKESLELEDPSSGLGVTKQDLGPVPM
+	6.307	−9.676	KPVSKMRMATPLLMQALP		+	5.263	−4.609	KPVSKMRMATPLLMQA
	1.406	−1.669	KPPKPVSKMRMATPLLMQA					
	0.778	−3.627	KPPKPVSKMRMATPLLMQAL					
	2.028	−3.301	KPPKPVSKMRMATPLLMQALP					
	2.374	−5.947	KPPKPVSKMRMATPLLMQALPM					
	1.634	−4.517	KPVSKMRMATPLLMQA					
	3.555	−3.864	KPVSKMRMATPLLMQAL					
	0.000	0.000	LPKPPKPVSKMRMATPLLMQAL					
	1.214	−7.773	LPKPPKPVSKMRMATPLLMQALPM					
	0.836	−2.487	RMATPLLMQALPM					
	0.000	0.000	MKLPKPPKPVSKMR		n.d.			
	0.488	−0.583	PSSGLGVTKQDLGPVPM		n.d.			
	0.980	3.583	LTVTSQNLQLENLR		n.d.			
	0.415	1.473	DVKIYLDENYERIN	Q9NQX7 Integral membrane protein 2	+	3.418	1.449	KIYLDENYERIN
	0.000	0.000	DVKIYLDENYERINV					
	0.196	1.133	DVKIYLDENYERINVP					
	0.837	−0.961	EDVKIYLDENYERIN					
	0.379	1.918	EDVKIYLDENYERINV					
	0.428	−3.087	EDVKIYLDENYERINVP					
	0.702	1.292	EDVKIYLDENYERINVPV					
	0.814	3.644	EDVKIYLDENYERINVPVP					
	0.035	−0.092	EDVKIYLDENYERINVPVPQ					
	0.209	−1.055	EDVKIYLDENYERINVPVPQFG					
	0.600	3.035	EEDVKIYLDENYERIN					
	0.835	2.703	EEDVKIYLDENYERINVP					
	1.175	4.143	EEDVKIYLDENYERINVPV					

*Comparison of the log2-fold change enrichment (DMH-DML) and the corresponding −log(*P*-values) for each peptide (left) or epitope (right) of three protein entries with highly abundant peptides. Uniprot accession codes of the protein sources are indicated as well as the original sequence of the peptides and the corresponding epitope provided by peptide landscape antigenic epitope alignment utility (PLAtEAU)*.

*Diff, stands for the difference of the log2-transformed intensity values and the −log(*P*-value) was estimated using a *t*-test considering all samples for each condition*.

Asterisks (*) indicates that the epitope arises from the overlap of two potential register-shifted epitopes and indicates the position (N- or C-terminal) of the “jump” (see Figure 1).

Using NetPanMHCII the binding cores and relative affinities for each peptide or epitope enriched for each condition were determined (Figure 6B, included in Tables S4 and S5 in Supplementary Material). Seqlogos were generated for each set of enriched peptides or epitopes and indicate a clear predominance of Asp in P4 in the core epitopes under all conditions. Binding affinity prediction using NetPanMHCII suggest that increasing DM expression results in lower levels of peptides classified as N.D. and an increase of predicted high-affinity binders. Such an observation is consistent with the expected function of DM as a peptide-exchange catalyst. The GO Term analysis for the peptides or epitopes enriched for each condition was done according to GO-Slim terms and modified as described in Section “Materials and Methods.” Our analysis suggests that increasing DM expression levels reduce the number of protein sources associated with membranes (primarily the plasma membrane), while increase the protein sources annotated in organelles including the Golgi apparatus, endoplasmic reticulum, and endosomal compartments (Figure 6C). It is worth noting that such a trend is more evident at the peptide level, where increased DM expression results in a complete removal of the “membrane” GO term. At the epitope level, on the other hand, such a trend in annotated GO terms is less prominent, and in case of the “membrane” term, it is only slightly reduced (10 to 8%).

Given the importance of DM expression in the display of epitopes related to autoimmunity (19), we inspected whether any autoimmune-related epitopes were found in our dataset, and how DM expression influences their display (Table 4). High expression levels of DM increase three previously reported autoimmune-related epitopes from the cytoplasmic actin 1 (actin B; epitope: AEREIVRDIKEKL), the GTP-binding nuclear protein Ran (epitope: APPEVVMDPALAAQYEH), and from the Lysosome-associated membrane glycoprotein 1 (LAMP1; epitope: LNTILPDARDPAFK) (32–34). When HLA-DM expression is relatively low, epitopes from the ubiquitous autoimmune antigens Syntenin-1 (epitope: LEDLKVDKVIQAQTA) and Calsyntenin-1 (epitope: DPPLIALDKDAPLRFA) are overrepresented (34, 35). Finally, several epitopes from autoimmunity-related antigens are overrepresented when HLA-DM is entirely absent, such as the epitope GTKVVLDDKDYFLFR from the mitochondrial heat-shock protein HSPE1 and ITSIVKDSSAARN from Syntenin-1 (33, 34).

**Table 4:** Enrichment behavior of peptides and epitopes of autoimmune-related antigens.

MaxQuant-peptides				Uniprot accession	PLAtEAU-epitopes			
Sig.	−log(*P*)	Diff.	Sequence		Sig.	−log(*P*)	Diff.	Sequence
	0.587	−2.197	DPPLIALDKDAPLRFA	O94985 Calsyntenin	+	2.502	−3.742	DPPLIALDKDAPLRFA
	1.124	−3.665	ENDNTVLLDPPLIALDKDAP					
	1.208	−4.413	ENDNTVLLDPPLIALDKDAPL					
	0.467	−0.721	NDNTVLLDPPLIALDKD					
	1.994	−4.383	NDNTVLLDPPLIALDKDAP					
	1.925	−4.465	NDNTVLLDPPLIALDKDAPL					
	0.112	−0.461	GKITSIVKDSSAARN	O00560 Syntenin-1		0.389	0.086	ITSIVKDSSAARN
	0.460	−0.509	GKITSIVKDSSAARNG					
	1.419	−1.538	GKITSIVKDSSAARNGL					
	0.053	0.065	GKITSIVKDSSAARNGLL					
	0.137	−0.620	ITSIVKDSSAARN					
	1.535	−5.911	ITSIVKDSSAARNG					
	1.350	−1.670	ITSIVKDSSAARNGL					
	0.538	−0.419	ITSIVKDSSAARNGLL					
	0.138	−0.539	KITSIVKDSSAARN					
	0.099	−0.137	KITSIVKDSSAARNG					
	0.763	−1.545	KITSIVKDSSAARNGL					
	0.039	0.050	KITSIVKDSSAARNGLL					
	0.029	−0.044	NGKITSIVKDSSAARNG					
	0.042	0.051	NGKITSIVKDSSAARNGLL					
	0.268	−1.424	SIVKDSSAARNGL					
	1.746	−4.972	LEDLKVDKVIQAQTA		+	3.230	−5.318	LEDLKVDKVIQAQTA
+	4.777	7.012	APPEVVMDPALAAQYEH	P62826 GTP-binding nucl. Protein Ran	+	8.732	7.461	APPEVVMDPALAAQYEH
	0.460	1.769	APPEVVMDPALAAQYE					
	1.716	3.894	APPEVVMDPALAAQYEHD					
	0.165	−0.158	AAQGEPQVQFK		n.d.			
	0.116	−0.387	KKNLQYYDISAK		n.d.			
	0.020	−0.084	AEREIVRDIKEKL	P60709 Actin B	+	1.776	2.580	AEREIVRDIKEKL
	1.255	4.053	AEREIVRDIKEKLCYV					
	0.968	−0.924	DDDIAALVVDNGSGMCK			0.000	0.000	DDDIAALVVDNGSGMCKA
	1.940	−4.570	DDDIAALVVDNGSGMCKA					
	0.256	−0.882	DDDIAALVVDNGSGMCKAG					
	0.049	0.288	DDDIAALVVDNGSGMCKAGFAGDDAPR					
	1.105	−0.425	AGFAGDDAPR		n.d.			
	0.123	0.760	AGFAGDDAPRAVFPSIVGRP					
	0.162	1.044	AGFAGDDAPRAVFPSIVGRPR					
	1.038	−2.015	GQKDSYVGDEAQSK		n.d.			
	1.439	−0.739	HQGVMVGMGQKDSYVGDEAQSK					
	0.285	−1.180	IVGRPRHQGVMVGMGQKDSYVGDEAQSK					
	0.412	1.787	MGQKDSYVGDEAQSK					
	0.218	−0.128	DSYVGDEAQSK					
	0.403	−1.718	SYELPDGQVITIGNER		n.d.			
	0.000	0.000	SYELPDGQVITIGNERF					
	0.761	3.078	SYELPDGQVITIGNERFR					
	0.272	−1.106	MQKEITALAPSTMK		n.d.			
	0.541	−0.306	EITALAPSTMK					
	0.083	−0.418	DLYANTVLSGGTTMYPGIADR		n.d.			
	0.053	−0.476	EIVRDIKEKL		n.d.			
	0.936	4.532	VAPEEHPVLLTEAPLNPK		n.d.			
	1.921	5.279	LNTILPDARD	P11279 LAMP1	+	4.065	1.838	LNTILPDARDPAFK
	0.155	0.853	LNTILPDARDPAF					
	0.028	0.039	LNTILPDARDPAFK					
	1.909	5.834	LNTILPDARDPAFKA					
	0.276	1.433	QLNTILPDARDPAFK					

*Comparison of the log2-fold change enrichment (DMH-DML) and the corresponding −log(*P*-values) for each peptide (left) or epitope (right) of three protein entries with highly abundant peptides. Uniprot accession codes of the protein sources are indicated as well as the original sequence of the peptides and the corresponding epitope provided by peptide landscape antigenic epitope alignment utility (PLAtEAU)*.

*Diff. stands for the difference of the log2-transformed intensity values and the −log(*P*-value) was estimated using a *t*-test considering all samples for each condition*.

*n.d. stands for not determined, in most of the cases peptides did not pass either the replication filter (two out of three replicates), the removal of background binders or any of them*.

## Discussion

Mass spectrometry has long been established as a suitable method to analyze MHC immunopeptidomes in both a qualitative and a quantitative manner (8). While most quantitative MS-based approaches have so far focused on MHCI, in the case of MHCII immunopeptidomes the situation is substantially less advanced. A key problem faced by any MS-based quantification strategy for MHCII-antigen presentation is the lack of uniform length of the eluted peptides. To circumvent this issue, Lippolis et al. (17) made use of manual alignment and relative intensities based on the total ion current to quantify 14 different sets of nested peptides from the immunopeptidome of DR4 molecules. More recently, Bergseng et al. (13) used the LFQ analysis facilitated by MaxQuant, based on the integration of the area under the curve, to estimate the relative abundance of peptides isolated from DQ molecules. The PLAtEAU analysis described here combines features of both approaches to rigorously and relatively rapidly achieve high-throughput quantification of MHCII-displayed immunopeptidomes. Since two residues on the flanking regions of the binding epitope (nine residues) presented by MHCII molecules have implications for their affinity (15) and immunogenicity (36–38), the grouping of nested peptides must be carefully interpreted, especially peptides of lengths between 11 and 13 amino acids. Interestingly, in this report we have shown that the grouping of peptides by PLAtEAU captures the original sequence of the nested peptides, with average deviation toward the N- or the C-termini of the sequences below two amino acids. On the other hand, such a strategy should provide a valuable tool for the investigation of antigen processing and presentation mechanisms. We designed and implemented a bioinformatics algorithm based on a custom Python script to identify sets of nested peptides and retrieve quantitative information about their abundance on the cell surface. The algorithm aligns peptides to their protein sources and identifies potential register shifts, taking into account the offset between the N-termini of the aligned peptides and the overlap between peptides aligning to the same region of a protein. The number of sets of nested peptides identified by this frame-shift feature is slightly higher for the DQ dataset (267/2,806 = 9.5%) (13) than that of the DR3 samples (9/276 = 3.2%).

We next sought to define the influence of the expression levels of the peptide-exchange catalyst HLA-DM on the immunopeptidome associated with HLA-DR3. In the absence of HLA-DM, MHCII molecules accumulate at the cell surface with their binding groove occupied mostly with peptides derived from the invariant chain (Ii) called CLIP. Besides, T cell hybridomas restricted for particular peptides could not be stimulated when DM-deficient cell lines were primed with the corresponding full-length protein (39–41). The impact of HLA-DM at the immunopeptidome level has been analyzed on the HLA-DR4 (42) allotype as well as in several HLA-DQ alleles (43). In addition, the expression levels of HLA-DM within model and primary APC subsets differ, and this differential expression has been correlated to the degree of antigen presentation of each cell subset (31, 44). HLA-DM is downregulated in rheumatoid arthritis patients when compared with controls (39, 45), and low HLA-DM expression levels seem to favor the presentation of collagen II-derived epitopes related to the pathogenesis of this disease (40, 46). Increased HLA-DM expression, on the other hand, has been correlated with an improved prognosis for breast cancer patients (41, 47). In sum, the level of HLA-DM expression has a clear impact on the composition of antigens presented to T cell hybridomas by APCs, and dysregulation of this expression can lead to the development of disease (40, 46). Using stable single clones expressing different levels of DM, we could show that the CLIP surface display is inversely proportional to HLA-DM expression levels. In addition, the relative amount of SDS-stable dimers increases with HLA-DM expression levels. Finally, the expression levels of HLA-DM impact the composition of the immunopeptidome associated with HLA-DR3 molecules. Our study reveals that, upon distinct HLA-DM expression levels, the display of endogenous epitopes other than CLIP also follows specific trends (increased or decreased presentation). Considering different expression levels of HLA-DM in distinct APC subsets (44), it is also likely that the immunopeptidome presented by each subset type will be have distinct signatures, that are more readily de-convoluted by PLAtEAU.

The two different DM conditions (low and high expression) yield clearly overlapping immunopeptidomes, with sets of specifically quantified or enriched peptides/epitopes. The high sensitivity and throughput of current shotgun proteomic approaches allows detection of large numbers of peptides present in only low abundances. To date, most of the immunopeptidome studies rely on peptide identifications based on the criteria of the FDR, which is usually set with a threshold value of 0.01. Many of these studies are focused on the identification of antigenic peptides and do not consider technical and biological replication. Our peptide identification criteria capitalize on the use of such replicates, and therefore identifications not matching our criteria are considered missing values. Statistically, there are three types of missing values in any proteomic dataset: missing completely at random (MCAR), missing at random (MAR), and missing not at random (26). MAR values are often considered in proteomics as MCAR missing values, which indeed account for peptides that have not been detected due to very low abundances, and they are expected to affect all measurements in an unbiased manner when the appropriate experimental set up is designed and used. On the other hand, MCAR represents the peptides that are below the detection limit, which is often understood as their absence from a sample. Another challenge of MS-based experimental approaches has to do with the fact that low sensitivity often translates into a large number of false positives. We sought to compensate the potential exclusion of peptides from the final dataset by allowing the identification software to include peptides matched by their mass and their elution profile (the “match between runs” feature of MaxQuant). Including an IP control for detection of background contaminants reveals that there is a large proportion of peptides that are enriched during the IP in the absence of HLA-DR and should therefore be considered as false positives. Our findings suggest that, despite stringent washing steps, unspecific binding to the matrix should always be considered. Strict biological and technical replication coupled with the use of IP controls are rarely reported in MHCII-immunopeptidome studies, but we clearly show that it is of particular relevance when trying to identify and quantify peptides with very low abundances. In total there are 1,011 unique peptides that are found in only 1 out of 3 replicates, and 821 unique peptides that are found in 2/3 technical replicates but are not found in both biological replicates.

Our aim was to introduce an algorithm allowing for the LFQ of MHCII immunopeptidomes. In this particular regard, we can clearly see that integration of MS1 values belonging to the same series of nested peptides into a total ion intensity that can be further normalized to the overall MS1 intensity of each run yields reliable results, as exemplified by the display of CLIP, one of the very few examples for which there are antibodies available against a particular MHCII–peptide complex. A similar approach has already been used (17) based on the fact that peptides belonging to such nested series often have similar, but not necessarily identical, ionization properties during MS/MS analysis. Grouping of peptides sharing a consensus epitope by PLAtEAU reveals that there are certainly important differences for the display of consensus peptides depending on the DM expression level. In a more general sense, the PLAtEAU analysis allows a robust determination of consensus epitopes of a typical length of 11–25 amino acids which are longer than the core 9mer sequences typically considered. While this type of analysis defines similar overall sequence preferences for a particular MHCII allotype (Asp in P4 for DR3), there are also notable differences between the sequence requirements calculated from peptide or epitope alignments (Seqlogos in Figure 6B). At this time it is hypothetical whether current binding affinity prediction tools could be improved by PLAtEAU and more quantitative binding data on peptides predicted by epitope analysis are required. In addition to a conceivable combination with prediction tools, PLAtEAU provides additional information on optimal peptide length and register-shifted epitopes. Thus, it will be a useful tool when defining optimized peptides in studies that capitalize on MHCII-antigen stimulation to elicit T cell responses.

The core epitopes identified in this study reveal a number of interesting new insights into the HLA-DM-mediated HLA-DR3-presented immunopeptidome. For one, we have introduced an additional control for removing background-binding peptides during the IP procedure. This allows for the filtering of epitopes arising from these background peptides, providing a higher-confidence quantification of the immunopeptidome. For example, in this background pool were epitopes from four different MHCII molecules (two from HLA-DRA, one from HLA-DRB, and one from the gamma chain) that were previously reported to be antigenic (13, 48). Included in this is an epitope of HLA-DRA (FGRFASFEAQGALANIA) that is frame-shifted from a previously reported antigenic HLA-DRA epitope (EAQGALANIAVDKAN) (13, 48). Furthermore, two epitopes from glyceraldehyde-3-phosphate dehydrogenase, which were previously reported to be antigenic, were also found among the background-binding epitopes in this study. The inclusions of epitopes of these previously reported antigens in the IP control suggests caution must be taken in the evaluation of immunopeptidome data, as even with stringent washing, false positives may result. In addition, quantitative differences in HLA-DR3 antigen presentation, including autoimmunity-related peptides depended on the expression level of HLA-DM as revealed by PLAtEAU analysis. This indicates that our method of analysis unfolds important antigenic features when analyzing disease-related immunopeptidomes. However, the versatility of PLAtEAU for the analysis of clinical proteomic data and cancer immunopeptidomes needs to come under scrutiny as it is encouraged with the script’s public release at the GitHub platform (https://github.com/e-morrison/plateau).

## Author Contributions

MA-B and EM conceived the research, performed the experiments, and analyzed the data with input and support from CF, EA, and BK. EM wrote the Python script. MA-B, EM, and CF wrote the manuscript.

## Conflict of Interest Statement

The authors declare that the research was conducted in the absence of any commercial or financial relationships that could be construed as a potential conflict of interest.
