# Supplementary material for: Quantification of HLA-DM-Dependent Major Histocompatibility Complex of Class II Immunopeptidomes by the Peptide Landscape Antigenic Epitope Alignment Utility
Source: Front Immunol. 2018 May 3;9:872. doi: 10.3389/fimmu.2018.00872 (PMC5943503; doi:10.3389/fimmu.2018.00872)
Supplement: Supplementary file 6 [file presentation_1.PDF]

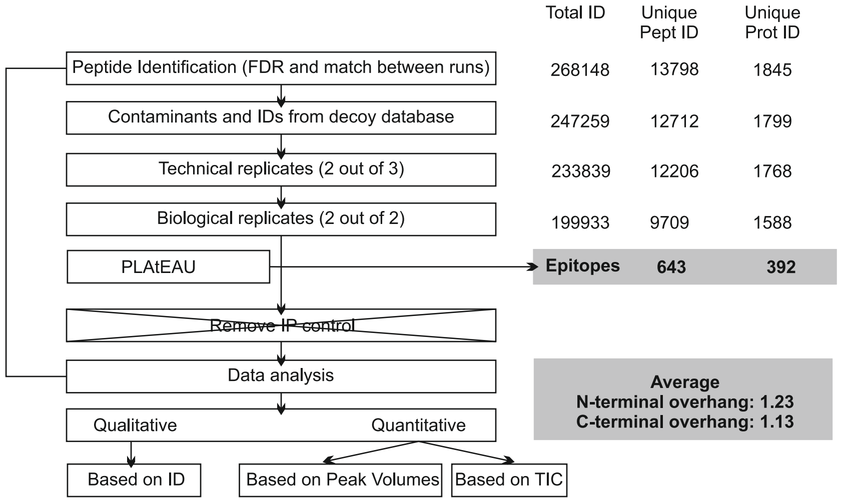

Figure S1. Benchmarking scheme for the HLA-DQ dataset published by Bergseng et al. 2015 (Ref. 13). A. Criteria stablished for peptide identification and its consideration for downstream processing is stated. Known contaminants (identified by an in-built database in MaxQuant) and identifications from the decoy database were removed at first. The resulting dataset is used for comparison with our approach. For PLAtEAU analysis we applied two extra filters including one for technical and another one for biological replication. This data set was used on the PLAtEAU script. In this case the peptides identified in the IP control (crossed box) could not be removed since the dataset does not include such a control sample. The number of identifications, peptides and protein sources are shown on the right side.

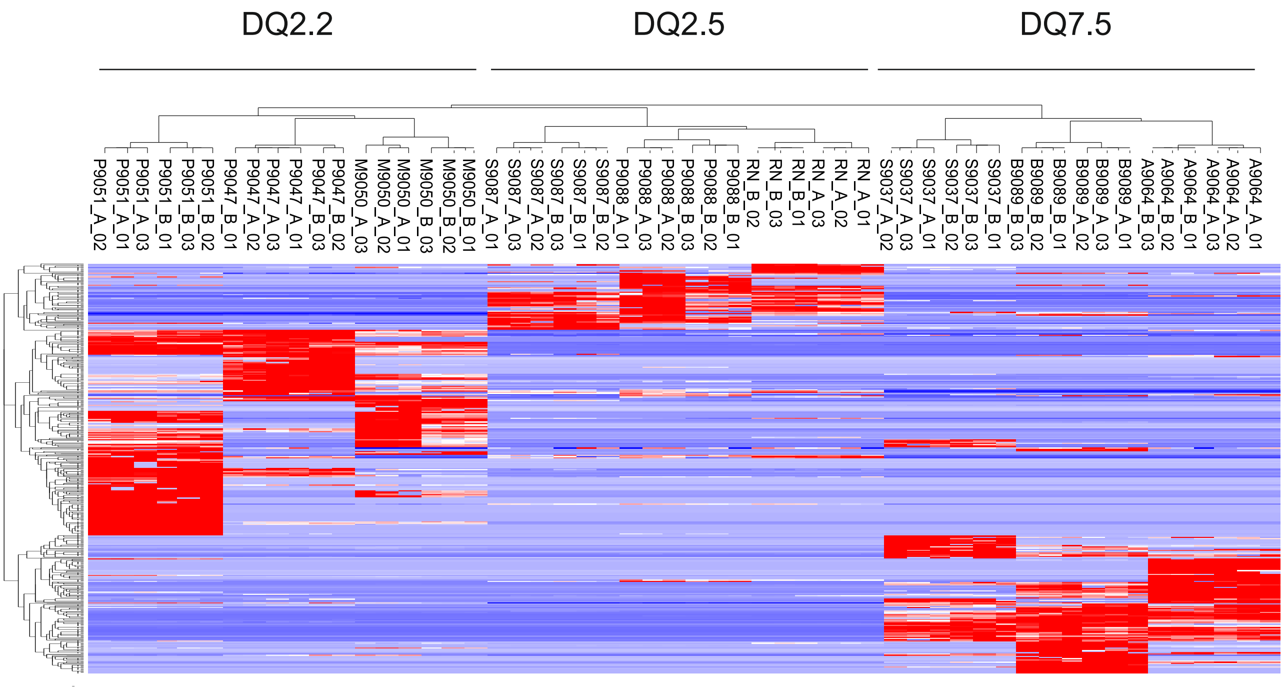

Figure S2. Heat map representation of the immunopeptidome published by Bergsen et al. 2015 (Ref. 13) using PLAtEAU. Hierarchical clustering based on LFQ intensity values retrived by PLAtEAU. Sample's name are kept as originally described. The HLA-DQ allotype expressed by the specific cell lines is shown on the top.

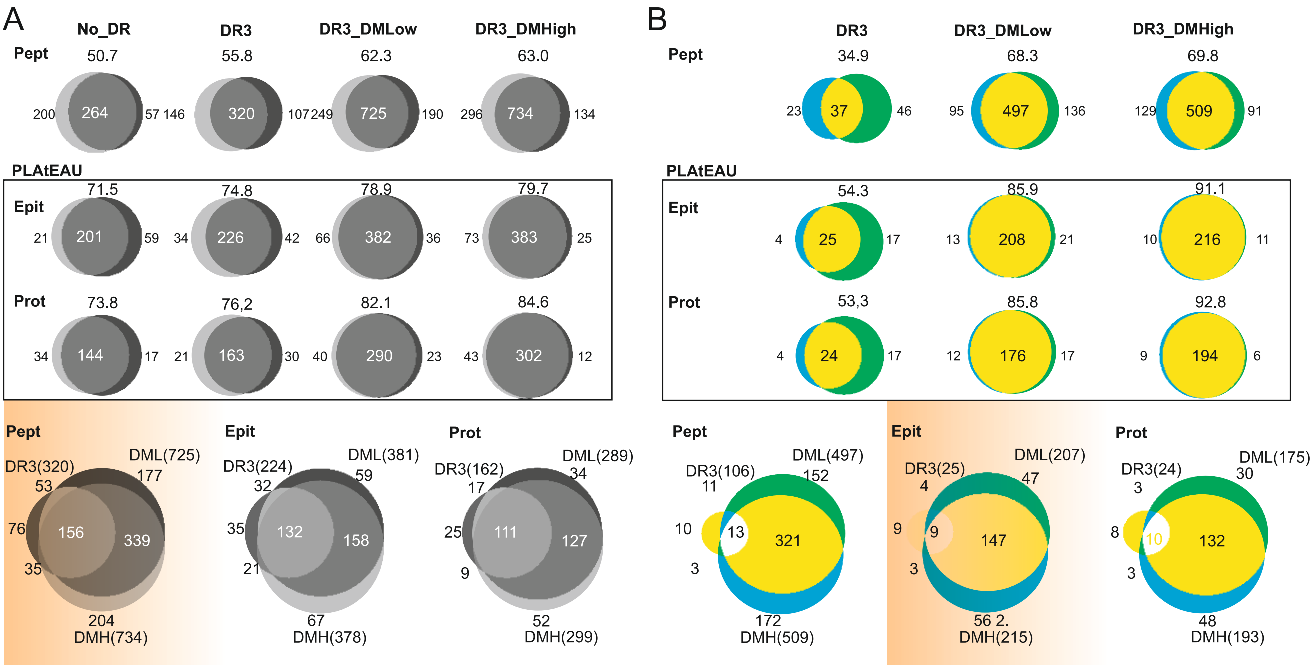

Figure S3. Overview of the peptides eluted from T2-DR3 cell lines. A. Venn diagrams showing the overlap between the different biological replicates used for each sample (above) at three different levels: peptide (identified directly from MaxQuant), consensus epitopes (defined by PLAtEAU), and unique protein entries derived from the consensus epitopes found by PLAtEAU. Venn diagrams of the overlapping immunopeptidomes of the three relevant samples at the same three different levels: peptides, consensus epitopes and protein sources. The overlap between biological replicates is indicated as number of proteins peptides or epitopes as well as in per cent. **B**. Same as in (A) after removal of the background peptides.
